# Conducting a phase III clinical trial in children during the COVID- 19 pandemic: Experience and lessons learnt from a clinical research facility of Nepal

**DOI:** 10.1080/21645515.2023.2239680

**Published:** 2023-08-04

**Authors:** Ram Hari Chapagain, Jessica Maharjan, Santosh Adhikari, Prabhat Thapa, Kshitij Kunwar, Bishnu Rath Giri, Nisha Jyoti Shrestha, Anil Kumar Shrestha, Sanjeet Kumar Shrestha, Suresh Man Tamang, Haeun Cho, Eun Lyeong Park, Jiyoung Lee, Jinae Lee, Deok Ryun Kim, Jae Seung Yang, Tarun Saluja, Anh Wartel, Julia Lynch, Katerina Rok Song

**Affiliations:** a OCV-S Trial Site, Kanti Children’s Hospital, Kathmandu, Nepal; b Department of Paediatrics, National Academy of Medical Sciences (NAMS), Kathmandu, Nepal; c Biostatistics & Data Management Unit, International Vaccine Institute (IVI), Seoul, Korea; d Science Unit, International Vaccine Institute (IVI), Seoul, Korea; e Clinical, Assessment, Regulatory, Evaluation (CARE) Unit, International Vaccine Institute (IVI), Seoul, Korea; f Europe Regional Office Director, International Vaccine Institute (IVI), Seoul, Korea; g Cholera Program Director, International Vaccine Institute (IVI), Seoul, Korea

**Keywords:** Clinical trial, COVID-19, experience, Nepal, pandemic

## Abstract

Clinical trials in humans are vital to test safety and efficacy of new interventions and are accompanied with the complexity of related regulatory guidelines, stringent time frame and financial burden particularly when participants are children. Conducting clinical trials in low and middle income countries, where 90% of global diseases occur, increases the complexity as resources, infrastructures, and experience related to clinical trials may be limited in some countries. During the COVID-19 pandemic, due to multiple infection control measures such as social distancing, lock-down of the societies, and increased work load of hospital workers, conducting clinical trials seemed very challenging. Related guidelines and recommendations on clinical trials required updates to adapt the situation for ongoing clinical trials to be continued and new clinical trials to be initiated. In this review report, we described the lessons learnt through our experiences, challenges we faced, and the mitigation measures implemented as a response while conducting a phase III clinical trial on a non-COVID-19 vaccine at a government children’s hospital during the COVID-19 pandemic. We hope this report will contribute in lowering the obstacles to allow the successful completion of future studies, in countries where people live with the burden of vaccine-preventable diseases.

## Introduction

A cluster of unexplained 27 pneumonia cases of unknown etiology were reported on December 31, 2019, which was later called as COVID-19. This slowly spread to all over the world and the outbreak was declared a pandemic on March 11, 2020, by the World Health Organization.^1,2^ The pandemic warranted several restrictions on all domestic and international travel, imposed social distancing in all public places and nation-wide lockdowns were implemented as mitigatory measures to control the rising number of cases. No country was exception to the pandemic, and in particular the impact on low- and middle-income countries like Nepal were devastating with the scarcity of medical supplies as well as health care resources that the pandemic exposed the country’s shortcomings in the health sector.

The clinical research sector was also significantly affected as the focus shifted to COVID-19 patients therefore, other ‘non-essential’ sectors were deprioritized or halted during the peak of the pandemic. The situation was exacerbated with the study site closures, and participants were hesitant to visit hospitals where COVID-19 patients are and the government order to stay at home.

Effective management of clinical trials is one of fundamental parts for successful the completion of clinical trials, not only to save its high running cost and timeline, but also to overcome the site specific practicalities. The challenges to conduct clinical trials differ from country to country and are often overlooked or underestimated by a sponsor during the early planning phase.^3^

In 2019, before the advent of COVID-19 pandemic, Nepal faced logistical challenges and a shortage of clinical trial experienced human resources, which required extensive planning, preparation, and training to set up a clinical trial site in the country.^4^ The challenges have significantly increased during the COVID-19 pandemic particularly before the introduction of COVID-19 vaccination as there was an increased shortage of human resources to manage the trials.

It is recommended that the feasibility and immediate necessity of starting a new clinical trial should be critically assessed by sponsors, in close collaboration with other relevant parties, in particular the investigators as the government was implementing stringent restrictions, rules and regulations barring to essential services only. The new rules on national and international level were updated to assess the risks and benefits section and the proper mitigation measures need to be addressed.^5^ This will be applicable to all type of pandemics.

In regard to the COVID-19 pandemic, Mitchell EJ et al.^6^ explained that the trial management team faced new uncertainties of re-starting clinical trials and it was unclear how it would move forward. The clinical trial teams had to work closely together to ensure their trials have adapted the evolving situation quickly, whilst ensuring study participant’s safety giving the utmost importance.

This paper aims to highlight the overall experience and challenges we faced while conducting a new clinical trial on a non-COVID vaccine, at a government children’s hospital during the COVID-19 pandemic. We believe the mitigation measures we implemented, will help other clinical trial managers and sponsors to design mitigation plans to complete clinical trials successfully when faced with similar situations in the future.

## Material and methods

A study titled ‘A phase III, Multicenter, Observer-blinded, Randomized, Active Controlled Trial to Evaluate Immune Non-inferiority, Safety and Lot-to-Lot Consistency of Oral Cholera Vaccine Simplified Compared to Shanchol^TM^ in 1–40 years old healthy Nepalese Participants,’ was planned with a sample size of 2530 in Nepal. The test vaccine was consisted of formalin inactivated *Vibrio cholerae* O1 Inaba Phil 6973, EI Tor and O1 Ogawa Cairo 50, Classical biotypes. The participants were divided into three age strata: 1–5 years old, 6–18 years old and 18–40 years old. This study was conducted at four sites in Nepal over a period of 20 months. This manuscript will focus on experiences from Kanti children’s hospital site where we recruited and followed 632 children from age strata 1 year to 14 years, from October 2021 to June 2022, after securing all required regulatory and ethical approval in Kanti children hospital and Nepal.^7^

This study was sponsored by International Vaccine Institute (IVI) and the vaccine manufacturer, EuBiologics Company limited in Korea. The primary objectives were to establish non-inferior immunogenicity of OCV-S to Shanchol™ in healthy children and adults, and to assess the safety profile of OCV-S. The study intended to compare the proportion of participants showing seroconversion against *Vibrio cholerae* O1 Inaba and Ogawa 2 weeks after the second dose of OCV-S to Shanchol™ for 1–40 years old. Safety of OCV-S was evaluated and described in each age stratum at 7 days and 28 days after each dose.

All potential trial participants were pre-screened by verbally asking a few questions, to exclude those who were ineligible to participate in the trial. After signing the Informed consent form (ICF) and informed assent form where required, the participants were then examined and checked for all inclusion and exclusion criteria. After randomization, blood samples were collected from all eligible participants, followed by the vaccine administration as per the randomization number generated via computer. The participants were then kept in observation for at least 30 minutes and were reexamined for any adverse events occurred. Diary cards were provided to the participants along with proper instructions on how to record the required information and appointment dates for the next visit was given. Each participant had a total of three site follow-up visits and four safety follow ups via telephone call. The final site visit was to be performed 182 days after the first dose of vaccine. Additional Standard Operating procedures (SOPs) on travel and the management of COVID-19 infected participants were prepared and implemented along with other essential SOPs related to the vaccine clinical trial. The potential bias has been limited by sharing the experience in group consensus as there might be bias in individual level. The factors (planning, regulatory approval process, site preparation, recruitment strategy, visit management, Investigational product (IP) management, sample storage and transport, and communication) affecting the successful completion of clinical trial are observed and described. The experiences have been described without using statistical methods.

## Experience of trial

### Preparation for clinical trial

International Vaccine Institute (IVI) based in Seoul, Republic of Korea has contributed in developing proper clinical research sites in Nepal, with the aim of promoting research culture and tapping into the enormous potential in less experienced settings by capacity building in developing countries. In collaboration with IVI, a vaccine trial on typhoid had been conducted in Nepal including Kanti Children’s Hospital. Following the completion of this typhoid vaccine trial, IVI proposed a vaccine trial for cholera (clinical trial-NCT04760236), an endemic disease in the local population.^7^

As the cholera vaccine trial was conducted during the COVID-19 pandemic, adequate space had to be secured to ensure proper social distancing. The infrastructure was modified by expanding the waiting lobby. Separate rooms were made available for consenting and examining the enrolled participant, blood collection, vaccine administration, post-vaccine observation and data entry, as required by the new guidelines implemented by the national governing bodies. Social distancing was maintained in all the rooms, with each room equipped with hand sanitizers and wash basins. Only one participant with her or his parents was allowed in at a time in each room. All participants and their parent/s had to compulsorily wear appropriate masks throughout their time at the hospital. In addition, the use of masks, face shields and other forms of PPE, as per WHO guidelines, were mandatory for all site staff.

Periodic lockdowns and restriction in people’s movements, as imposed by government due to the high risk of transmission of COVID-19 infection in high-risk areas such as hospitals, where there was a possibility of overcrowding, were hindrances in ongoing trials and in trials scheduled to kick off soon. Also, a risk of acquiring COVID-19 infections among health care workers, clinical research staff and trial participants was a potential threat. Fortunately, the trend of daily COVID-19 cases was decreasing when the official enrollment at Kanti children’s hospital commenced in October 2021. Improvement in hand hygiene and sanitation might have decreased the number of diarrheal illnesses or other infectious diseases which in turn might have decreased the number of unscheduled visits for such illnesses.

We presumed our vaccine clinical trial would be of lower priority than the clinical trials for vaccines against COVID-19. Nevertheless, we were surprised to see that people from all walks of life and varying economic backgrounds were interested to participate in the cholera vaccine trial, in the midst of a pandemic. We ensured the participant to understood that the trial was not on a vaccine against COVID-19, as one may have mistakenly thought, in light of the pandemic situation. The media appeared to play an important role in raising awareness of the necessity of vaccines in general. The pandemic has led to greater global awareness of the role and importance of clinical trials in vaccine research and healthcare.^8^

### Regulatory and ethical consultations

The research trial was scheduled to start in August-September 2021; however, there were several delays due to concerns in the setting of the rising number of COVID-19 cases and high transmission rates. In order to obtain regulatory approvals before the commencement of the trial, the study team had multiple consultations with the Council of Ministers and the Ministry of Health and Population as per the newly enforced laws on conducting a vaccine related trials during an ongoing pandemic.^9^ The new law therefore lengthened with additional ‘steps’ that were required before the trial could be started.

### Staff recruitment and training

ICH-GCP requires all site staff to be trained ahead of the start of a clinical trial.^10^ Due to the pandemic, the first sessions of Good Clinical Practice (GCP) and Good Documentation Practice (GDP), project specific site initiation training was conducted virtually. As countries worldwide had imposed travel restrictions, the training was conducted via the next best alternative-virtual mediums, such as Microsoft Teams and Zoom, during June and July 2021. These virtual training sessions proved to be fruitful and convenient for both site staff and trainers, particularly for those who were restricted to travel from overseas.

The second sessions on the same topics were completed on-site a few months later (in late August 2021) when the COVID-19 cases were on a decreasing trend, with the daily cases averaging at about 1500, and the ban on international travel lifted. Site initiation training was also conducted in-person following all national guidelines on social distancing, in properly cross-ventilated rooms, with appropriate use of face masks and face shields. The combination of the initial virtual training and physical on-site training later on, proved to be an effective method. Limitations of virtual training sessions were covered by on-site and hands-on training, but the topics discussed in the virtual training significantly saved time during on-site sessions.

The site staff contracting COVID-19 infection were isolated at home and repeated with PCR testing as per the WHO guidelines. And study staff who was in contact with a person suspected of COVID-19 infection were quarantined for certain duration and be tested for COVID-19 PCR. Based on this situation, the training was extended to potential back-up staff making the number of trainees double the number of anticipated study staff that in case of any staff’s emergency medical leave, back-up personnel can immediately fill in the gap. This strategy also assisted during times of heavy enrollments. Thus, for smooth running of the trial in the a pandemic setting, the training of adequate manpower should be kept in mind.

We also ensured that all site staff members were fully vaccinated with the vaccine available at that time. Also, it was ensured that all site staff members were kept up to date regarding the guidelines on quarantine and isolation for COVID-19. Thus, ensuring COVID-19 vaccination of study staff and strictly following the latest guidelines on COVID-19 helped ensuring minimizing the gap in study staff.

### Recruitment of trial participants

The number of participants approached were higher than we previously experienced in another clinical trial before COVID-19 pandemic, as we had mechanisms of communicating the information about the clinical trial being conducted in Kanti Children’s hospital via different stake holders, such as the local health institutions owned by government or community level, and they were called to the trial site on appointment basis which ensured the crowd control.

### Pre-screening for COVID-19 at site

As an emergency response to the pandemic, most hospitals in Nepal mandated screening for COVID-19 at the hospital entry. All visitors were required to have their temperature checked and were questioned about symptoms such as fever, cough, myalgia, and etc. On this basis, symptomatic patients were sent to a dedicated COVID-19 clinic which was set up for further evaluation of suspected COVID-19 cases. All potential participants, therefore, would have already had their temperature checked when they reached the trial site, which is located inside the hospital premises. However, as a precautionary measure, we recruited a dedicated staff, to check temperature and ask for any illness or relevant medical conditions at the entry point. COVID-19 vaccines for children had not yet been made available at the time of enrollment. We have high COVID-19 vaccine coverage showing no hotspot for vaccine hesitancy.^11^

### Informed consenting

The recruitment of participants started after decreasing trend was observed in the daily number of COVID-19 cases. The daily number of new COVID-19 cases was averaging at about 849 over a month, and the 7-day average was 779 on the first week of October 2021, when the enrollment commenced.^11^

Interested people, who enquired about the vaccine over the phone, were initially pre-screened and briefly given an explanation about the vaccine, to minimize cases that would ultimately have an absolute or a relative contraindication to the vaccine. After they confirmed their interest in the trial, they were given specific times for an appointment for formal informed consent, to reduce the time at the site and to avoid cross-exposure with other participants.

Crowd control may be especially difficult in the setting of studies involving children, as often, both parents come together with the child. This nearly triples the crowd in comparison to trials involving only adult participants. In the setting of the ongoing pandemic, our field mobilizers had a particularly important role to play in the smooth recruitment of participants, and to ensure a balanced number of enrollments every day. Work hours were extended and the site was opened in off hours to decrease the flow of people at any particular given time, which proved to be efficient in reducing exposure between participants. The site was also kept open on weekends and on public holidays so that our trial participants, who were children, would not miss their online academic classes. This required site staff to work overtime and they were compensated with adequate remuneration for extra hours. This increased the site personnel cost by 33.0%. There was also a concern regarding health insurance of staff, which would ultimately lead to increased cost, when compared to trials that were conducted before the pandemic. The lesson to be learnt from this trial involving children in the setting of a pandemic is that the trial sites should be flexible in work hours and keep the site open during off hours and holidays as per the need. Similarly, decisions about how to maintain enrollment during the pandemic and in the event of future similar disruptions must be prioritized.^12^

It was not feasible for the written informed consent to be performed online.^13^ Considering that a significant proportion of the trial participants were not familiar with online technologies, explaining all the trial and site visit procedures and receiving an electronic signature on the consent forms, in the end, would prove to be a tedious task despite easy internet accessibility. We assumed that this seemingly minor inconvenience would discourage potential participants from participating in the trial and we decided that consenting would be more fruitful if performed on site, to have a connection with the participant and to gain their trust.

Devising newer ways for virtual consenting with easy user interface would definitely prove to be fruitful in future trials taking place during a pandemic if the regulatory body of Nepal permits. After consenting, enrolling participants attend visits on an appointment basis, which will significantly decreases the time of hospital stay and aid crowd control.

### Physical examination

After signing the consent forms, the trial protocol mandated a general physical examination and vital signs check of the enrolled participant before collection of a blood sample and the administration of a vaccine. The examination of participants, the collection of first blood sample, vaccine administration and post vaccine examination were all performed on the same day. All enrolled participants were open to have the first dose of vaccine on the day of consenting to lessen one additional site visit, and an option to receive the vaccine on a later date, within one week of signing the consent forms as per the protocol, was also available.

After the administration of the vaccine, there was a 30 minutes observation period which overlapped for many participants. A designated spacious lobby just outside the vaccination room was used for the observation of the participants post-vaccination. Proper management had to be implemented to ensure proper crowd control, with adequate space for children to move around.

Fortunately, at our site, we were able to finish recruitment of participants before the third wave of COVID-19 (January 2022), therefore we were able to significantly minimize cross-exposure.

### Communication with site staffs and stakeholders

In the setting of a pandemic, the face to face communication between site staff, the sponsors and Clinical Research Organization (CRO) may be affected, especially in cases of multicentric clinical trials. Scanned copies of formal letters were sent via email.

We had to physically visit the office of National Health Research Council (NHRC), a regulatory body of research, to receive acknowledgments of various documents submitted to during the trial, which could have easily been replaced by providing“auto-reply” to the e-mail sent, or by establishing an ad-hoc web portal.

### IP management, personal data collection and blood sampling

Data collected at our site, such as anthropometric measurements like height, weight, age and vital signs and the collection of blood samples took longer than usual, due to concerns regarding the spread of infection. Data regarding safety was reported by the participants themselves at their homes and were submitted during subsequent visits through diary cards. Some parents with younger children showed concern about the amount and frequency of blood drawn. Fortunately, there were no study withdrawals due to frequency and amount of blood.

Regarding the transport of Investigational Products (IP) and serum samples, air and road routes were well defined, with back-up transport strategies in case of delay or the cancellation of flights to ensure smooth execution. Prior arrangements were made with World Courier services, to supply cold chain boxes capable of maintaining required temperatures for up to seven days which were used for serum samples and IP. Similarly, to ensure IP condition at sites, two sets of IPs; the main set and a back-up set were maintained at two different locations. The blood samples were stored, as per the protocol, and were sent to the lab in Korea by World Courier. There was a provision to keep the samples for a longer time if the air traffic was halted, but fortunately, we faced no such delays, and the process ran smoothly. The samples were transported to Korea as soon as we completed the sample collection process.

### Retention of participants and safety follow up

As provisioned by the protocol, safety follow ups could be done either by telephone calls or through home visits by a site staff. In our study, all safety follow ups were performed through telephone calls by a medically qualified study staff and home visits were completely avoided. As regular contacting and engagement is required to ensure retention of participants until the end of study, our site staff maintained all telephonic records as per the protocol, and efforts were made to ensure that participants, who could not be reached due to issues involving networks and others, were re-contacted. A dedicated study phone was used by the study team to ensure none of the calls were missed, and the trial participants could be in touch with a health care provider of the site 24/7. This is also recommended by Dong E et al.^14^ There should be flexibility in the time for phone contacts can be made, as guardians might be busy during the day in other works. Most of the parents were contacted in the evening or morning. This changed the research staffs regular working hours. Parents needed longer time to discuss their child’s condition, although they were informed about the information during the vaccination. We perceived that the telephonic follow-up conducted by health personnel is equally effective to pick up the adverse events, compared to the physical presence. This resulted in 100% follow-up rate at the final follow-up visit, which was a mandatory physical visit at the site as per the protocol.

In a pandemic situation, a flexible study protocol which allows various ways of follow ups of participants helps to minimize follow up loss or protocol deviations. We had only four protocol violations. There may be issues such as the child becoming sick which may cause many lost-to-follow-up cases. The dropout rate of our study was 7 out of 632 participants. There was a chance of protocol deviation if any of the participants contracted COVID-19. Fortunately, none of the study participants contracted COVID-19 during the study period. Also, the patient may receive other vaccines as well such as COVID-19 vaccine and Typhoid vaccines, as the government had started a mass campaign for vaccination.^15^

### Logistics management and technological supports

The logistics were provided by a local company contracted to our site. This made it easier for us as the local vendor was mobile to purchase trial related materials during the pandemic. This also shifted our focus more on enrollment and other clinical trial procedures.

### Monitoring and audit

Monitoring of the trial is possibly the most significant ongoing challenge for trials sites during the pandemic.^16^ Our study recruited one blinded internal monitor at the site; however, remote monitoring was also performed. Clinical research associates (study monitors) usually visit the site only during their dedicated monitoring periods, however, due to the pandemic, monitors stayed nearby the site throughout the enrollment and follow-up period, as they could not travel to and from their office due to travel restrictions. This increased the chance of providing onsite coaching to the site staffs and it increased the chances to observe the site practice of non-trial related procedures.

It was a challenge to continue the ongoing trial during COVID-19 pandemics. There are many factors those need to be considered and addressed to conduct new clinical trials during pandemic for a non-COVID-19 vaccine.

## Conclusion

In this report, we have shared the experience of conducting a non-COVID-19 vaccine clinical trial in Nepal, one of low and middle income countries, during COVID-19 pandemic. This study was conducted at the end of second wave of COVID-19 when most of adults have already been vaccinated and people have more ideas on how to combat COVID-19. There are some limitations of this report. We have shared the experience from a single site without determining the covariates nor applying statistical tools. The COVID-19 pandemic has resulted in insecurities and changes to the way in which clinical trials are conducted. Adjustments in trial site opening and closing time, lengthy regulatory clearances, the modification of physical infrastructure of trial sites, special consideration in preparing extra human resources, increased virtual communication from training to reporting and increased trial cost are the main factors and observations we observed during this trial.
